# Intuitionistic Fuzzy TOPSIS as a Method for Assessing Socioeconomic Phenomena on the Basis of Survey Data

**DOI:** 10.3390/e23050563

**Published:** 2021-05-01

**Authors:** Ewa Roszkowska, Marta Kusterka-Jefmańska, Bartłomiej Jefmański

**Affiliations:** 1 Faculty of Economics and Finance, University of Bialystok, 15-062 Białystok, Poland; 2 Department of Quality and Environmental Management, Wroclaw University of Economics and Business, 53-345 Wrocław, Poland; marta.kusterka-jefmanska@ue.wroc.pl; 3 Department of Econometrics and Computer Science, Wroclaw University of Economics and Business, 53-345 Wrocław, Poland; bartlomiej.jefmanski@ue.wroc.pl

**Keywords:** intuitionistic fuzzy sets, intuitionistic fuzzy TOPSIS, subjective quality of life, European cities

## Abstract

In the assessment of most complex socioeconomic phenomena with the use of multicriteria methods, continuous data are used, the source of which are most often public statistics. However, there are complex phenomena such as quality of life and quality of services in the assessment, for which questionnaire surveys and ordinal measurement scales are used. In this case, the use of classic multicriteria methods is very difficult, taking into account the way of presenting this type of data by official statistics, as well as their permissible transformations and arithmetic operations. Therefore, the main purpose of this study was the presentation of a novel framework which can be applied for assessing socioeconomic phenomena on the basis of survey data. It was assumed that the object assessments may contain positive or negative opinions and an element of uncertainty expressed in the form a “no”, “difficult to say”, or “no opinion” answers. For this reason, the intuitionistic fuzzy TOPSIS (IF-TOPSIS) method is proposed. To demonstrate the potential of this solution, the results of measuring the subjective quality of life of the inhabitants of 83 cities in EU countries, EFTA countries, the UK, the Western Balkans, and Turkey are presented. For most cities, a high level of subjective quality of life was observed using the proposed approach. The highest level of quality of life was observed in Zurich, whereas the lowest was observed in Palermo.

## 1. Introduction

Multicriteria decision making (MCDM) offers several techniques which are used in comparative analyses of complex socioeconomic phenomena [1,2]. The analysis concerns objects such as countries, regions, and cities which are characterized by a set of socioeconomic indicators. We can distinguish three main approaches used in multicriteria methods: utility function, outranking relation, and sets of decision rules [3]. To handle the problems of analyzing complex socioeconomic phenomena, the most useful methods are those based on a utility function, which allows aggregating information about objects into a unique parameter. These methods allow ranking objects and classifying them according to the values of aggregated measure.

The most popular methods based on the synthesizing criterion are SAW (simple additive weighting) [4], TOPSIS (technique for order of preference by similarity to ideal solution) [5], AHP (analytic hierarchy process) [6], and VIKOR (visekriterijumska optimizacija i kompromisno resenje) [7]. Choosing among these methods depends on the problem under consideration and the available data.

One of the most frequently used methods in practice because of its simplicity and rationality is TOPSIS, developed by Hwang and Yoon in 1981 [5]. The advantages and the usefulness of the TOPSIS in comparative analyses stem from the fact that the objects are compared to so-called reference points. In the classic variant of TOPSIS, the coordinates of the reference objects are based on the maximum and minimum values. In some studies, however, objects were compared to a specific object selected as a benchmark or to desired target values [8]. Examples include indicators for SDG, where a different target value is specified for each country.

The spectrum of socioeconomic analyses to which the TOPSIS method is applied is very wide. Studies using the TOPSIS method have investigated human capital [9], the macroeconomic situation [10], international trade performance [11], smart development [12], innovation [13], energy performance [14], sustainable development [8,15,16,17,18], economic development [19], social development [20], healthcare [21], and eGovernment development [22], among others. The authors used different variants or hybrids of TOPSIS methods, and their analyses were based on different sources of data.

Balcerzak and Pietrzak [9] assessed changes in the quality of human capital in EU countries. Stankovic et al. [12] prepared a ranking of central and eastern European cities, based on various elements of their smart performance, by combining the AHP (analytic hierarchy process) for determining the relative importance of criteria and the TOPSIS method of ranking. Piwowarski et al. [15] assessed EU countries in terms of sustainable development using the VIKOR and TOPSIS methods. Roszkowska and Filipowicz-Chomko [8] measured sustainable development in the area of education in EU countries using an extended TOPSIS procedure, which took into account EU targets and/or national targets in building a positive ideal solution and a negative ideal solution. Sielska [21] conducted a comparative analysis of the level of healthcare in European Union countries. Vavrek and Chovancová [14] analyzed the energy performance of EU member states concerning the priorities of the European Energy Union. The common feature of the above-presented surveys is that they were conducted using Eurostat databases.

In addition to Eurostat, there are other popular sources of data used in the analysis of socioeconomic phenomena for countries. Kaynak et al. [13] compared the innovation performance of EU candidate countries using an entropy-based TOPSIS. In the comparative analysis, they used information from the following reports: Global Competitiveness Index, Innovation Union Scoreboard, Knowledge Assessment Methodology (KAM), and Global Innovation Index. Eyüboğlu [10] determined the macroeconomic performance of developing countries using data such as economic growth, inflation rate, unemployment rate, and the current account balance/GDP published by the OECD and the IMF. The TOPSIS method was used for the ranking of performance, whereas AHP was used for the determination of criteria weights. Karabiyik and Kutlu [11] assessed the international trade performance of OECD countries on the basis of data from the World Trade Organization Statistics Database. Vavrek and Ardielli [22] evaluated eGovernment development in EU member states using data from the European Commission, Eurostat, and the United Nations.

Other popular sources of data in socioeconomic research are national or regional statistical yearbooks. Sustainable urban development in China was analyzed by applying the modified TOPSIS method and using different source data from the Regional Statistical Yearbook. Ding et al. [23] provided a comprehensive evaluation of urban sustainable development in China using the TOPSIS-entropy method. The data used in this study came from Chinese social and economic statistical yearbook databases, including the China Urban Construction Statistical Yearbook, China City Statistical Yearbook, and China Statistical Yearbook for Regional Economy. Li et al. [16] assessed the sustainability of cities in northeastern China using dynamic TOPSIS-entropy methods. The research data were extracted from the Liaoning Provincial Statistical Yearbook, Jilin Provincial Statistical Yearbook, Heilongjiang Provincial Statistical Yearbook, and China City Statistical Yearbook. Tang et al. [17] assessed the urban sustainability of selected cities in China using a modified TOPSIS based on gray relational analysis and data from the China City Statistical Yearbook, the Anhui Statistical Yearbook, and the Statistical Yearbooks of the cities. Łuczak and Just [19] explored two types of TOPSIS (classical and positional) for the assessment of economic development of units at different government levels. The study was based on data from the Central Statistical Office of Poland. Stecyk et al. [18] used the AHP/TOPSIS model in the analysis of the level of sustainable development regions in the West Pomeranian Province in 2010 and 2017 in Poland using data from the Statistical Office in West Pomeranian Province. Roszkowska and Filipowicz-Chomko [20] applied TOPSIS with a common development factor for the analysis of regional differentiation in the social development of Polish voivodeships in the context of sustainable development. The study used the data from the Central Statistical Office of Poland. Perło and Roszkowska [24] applied soft modeling and the TOPSIS method for the analysis of the competitiveness of companies in urban functional areas in Poland using data from the Central Statistical Office of Poland and the Customs Office.

The vast majority of the studies mentioned were based on criteria whose values were expressed on an interval and/or ratio scale. However, among complex socioeconomic phenomena, there are also those based on public opinion. Such phenomena include, for example, subjective quality of life, quality of public services, and air quality. Data for this type of analysis are collected, for example, by the European Commission within the framework of the international Eurobarometer public opinion survey project. Another source of data based on public opinion is one of the largest European research projects, the European Sociological Survey. As part of the research projects mentioned, the values of criteria are most often measured using ordinal scales (e.g., Likert scales). Measurement results are presented as percentages of responses to particular categories of ordinal measurement scales, and researchers often do not have access to raw data. Therefore, the comparability of the assessments of criteria is much more difficult. In such a case, multicriteria assessment using the TOPSIS method would require the multiplication of percentages of responses in particular categories by numerical signs assigned to categories of ordinal measurement scales. Such a procedure would make it possible to obtain average assessments of criteria for objects and use the classic TOPSIS method. In the literature, we found very limited propositions for how to deal with ordinal scales in survey data. Such a procedure based on the Hellwig approach was proposed by Jefmański [25].

Motivated by the presented studies, this article proposes a new approach based on the fuzzy intuitionistic TOPSIS method to deal with the problem of aggregating respondents’ opinions presented by official statistics. The contributions of the paper are as follows: firstly, we described the problem of analyzing survey data and building synthetic measures where data are represented on an ordinal scale. Then, the IF-TOPSIS based model for the evaluation of questionnaires was proposed. The novel IF-TOPSIS method has several advantages: (1) it does not require overstating the level of measurement or subjective fuzzy number parameters to be set; (2) the method allows taking into consideration not only positive and negative opinions about the objects in the questionnaires, but also “I do not know” or “no opinion” answers, i.e., hesitancy in the evaluation object. Ideal and nonideal points were used as stable benchmarks allowing for an independent evaluation of each object with respect to criteria. Importantly, our proposition avoids rank reversal, which can occur if objects are confronted with new information that was not considered when the evaluation process was performed [26]. Lastly, the classification of objects with respect to the IF-TOPSIS ranking is also proposed.

To illustrate the suitability of the proposed methodological approach to the resolution of real decision-making problems, we applied IF-TOPSIS to the ranking of European cities concerning quality of life in 2019. A comparison analysis with classical TOPSIS methods was conducted to show the advantages and limitations of the IF-TOPSIS method. Results indicate that the method is suitable and effective for solving problems with evaluating survey data.

The rest of the paper is organized as follows: the fundamentals of intuitionistic fuzzy sets are first presented, and the intuitionistic fuzzy TOPSIS approach, along with its applications, is highlighted. Next, we propose the novel framework of intuitionistic fuzzy TOPSIS to tackle the problem of assessing socioeconomic phenomena on the basis of survey data. Furthermore, we apply the proposed approach to an evaluation of cities using a dataset for the subjective quality of life of the inhabitants of 83 cities in EU countries, EFTA countries, the UK, the Western Balkans, and Turkey in 2019. Lastly, we present the conclusions of the paper, and future research is proposed.

## 2. Preliminaries

In this section, we briefly introduce some basic concepts related to intuitionistic fuzzy sets (*IFSs*) and distances on *IFSs*, which are used in the IF-TOPSIS procedure.

Zadeh [27] introduced the fuzzy set (*FS*) theory to deal with vagueness and uncertainty. One of the well-known extensions of *FS* is the intuitionistic fuzzy set theory (*IFS*) proposed by Atanassov in 1986 [28].

**Definition** **1.** *According to* [28,29], *let* X *be a universe of discourse of objects. An intuitionistic fuzzy set* A *in* X *is given by*

(1) $$A=\left\{<x,\;\mu_{A}(x),\;\nu_{A}(x)>|x\in X\right\},$$

*where* $\mu_{A},\;\nu_{A}:X\to [0,\;1]$ *are functions with the condition for every* x∈X.

(2) $$0\leq \mu_{A}(x)+\nu_{A}(x)\leq 1,$$

*where the numbers* $\mu_{A}(x)$ *and* $\nu_{A}(x)$ *denote, respectively, the degrees of membership and non-membership of the element* x∈X *to the set A;* $\pi_{A}(x)=1-\mu_{A}(x)-\nu_{A}(x)$ *represents the intuitionistic fuzzy index (hesitation margin) of the element x in the set A.*

If the universe *X* contains only one element x, then the *IFS* A over X can be denoted as $A=(\mu_{A},\;\nu_{A})$, which is called an intuitionistic fuzzy value (*IFV*) [30,31]. Let Θ be the set of all *IFVs*. Clearly, the fuzzy value (1, 0) is the largest, while (0, 1) is the smallest.

Some researchers [32,33,34] noted that intuitionistic fuzzy sets can reflect the decision-maker’s approval, rejection, and hesitations. Moreover, entropy was proposed by Zadeh [27] as a measure of fuzziness. For *IFSs*, different frameworks of entropy have been proposed by [35,36,37], among others. The definition introduced by Burillo and Bustince [35] allows us to measure the degree of intuitionism of an *IFS*. Szmidt and Kacprzyk [36] proposed a non-probabilistic-type entropy measure that is the result of a geometric interpretation of *IFSs*, which uses a ratio of distances between two of the *IFSs*. Hung and Yang [37] introduced the concept of probability for defining the entropy of an *IFS*. The concept of entropy and fuzzy entropy is widely used for determining weights in multicriteria methods.

Two well-known types of distances used for fuzzy sets are Euclidean and Hamming distances [33]. In the paper, we used the concept of Euclidean distance.

**Definition** **2.** *According to* [33], *let us consider two* A, B∈IFS *with membership functions* $\mu_{A}(x)$*,* $\mu_{B}(x)$ *and non-membership functions* $\nu_{A}(x)$*,* $\nu_{B}(x)$*, respectively. The normalized Euclidean distance is calculating as follows*:

(3) $$d\left(A,\;B\right)=\sqrt{\frac{1}{2n}\sum_{i=1}^{n}\left[{\left(\mu_{A}(x_{i})-\mu_{B}(x_{i})\right)}^{2}+{\left(\nu_{A}(x_{i})-\nu_{B}(x_{i})\right)}^{2}+{\left(\pi_{A}(x_{i})-\pi_{B}(x_{i})\right)}^{2}\right]},$$

(4) $$d\left(A,\;B\right)=\sqrt{\frac{1}{2n}\sum_{i=1}^{n}\left[{\left(\mu_{A}(x_{i})-\mu_{B}(x_{i})\right)}^{2}+{\left(\nu_{A}(x_{i})-\nu_{B}(x_{i})\right)}^{2}\right]}.$$

## 3. Intuitionistic Fuzzy TOPSIS (IF-TOPSIS) Method—Methodological Consideration and Application

One of the most well-known MCDM methods based on reference points is TOPSIS introduced by Hwang and Yoon [5]. In the TOPSIS approach, the most preferred alternative has the shortest distance from the positive-ideal solution and the farthest distance from the negative-ideal solution. TOPSIS has been applied to solve multicriteria problems because of the sound logic and rationale of human choice [38]. To handle several conflicting criteria for which the decision-maker’s knowledge is usually vague and imprecise, the TOPSIS method was modified into a fuzzy environment.

The recent developments of the TOPSIS technique [39,40] and fuzzy TOPSIS technique [41,42] were presented in review papers. One of the very popular variants of the fuzzy TOPSIS method, despite the fact that it was proposed relatively recently, is TOPSIS based on the concept of intuitionistic fuzzy sets.

Boran et al. [43] proposed intuitionistic fuzzy TOPSIS, where the ratings of alternatives with respect to criteria and weights were given as linguistic terms characterized by intuitionistic fuzzy numbers. They applied intuitionistic fuzzy TOPSIS for the evaluation of suppliers. In another paper [44], intuitionistic fuzzy TOPSIS was used in choosing the location of a new plant. Four potential sites characterized in terms of five evaluation factors were scored. Joshi and Kumar [45] developed an intuitionistic fuzzy TOPSIS method based on a distance measure and intuitionistic fuzzy entropy for multicriteria decision making. Büyüközkan and Güleryüz [46] proposed a method for evaluating and selecting mobile phones. In the presented example, they assessed three models of mobile phones characterized by six criteria. Three experts assessed the validity and performance of the criteria using nine- and six-label linguistic evaluation scales. Rouyendegh [32] proposed a hybrid model based on analytic network process (ANP) and intuitionistic fuzzy TOPSIS methods for the problem of uncertainty and subjectivity under certain environments, which were handled using linguistic values and weights via the ANP method. Wang et al. [38] presented an integrated OWA/TOPSIS approach in the intuitionistic fuzzy environment for solving MCDM problems. Zulqarnain and Dayan [47] proposed a method of supplier selection for an automotive company based on linguistic variables and IFT. Shen et al. [30] presented an extended IFT method based on a new distance measure to evaluate a potential strategic partner’s credit risk. Five potential strategic partners were assessed against five criteria. Rouyendegh et al. [48] applied intuitionistic fuzzy TOPSIS to solve the problem of wind powerplant site selection. Chen [49] proposed a multicriteria assessment model which combines intuitionistic fuzzy entropy-based TOPSIS with grey relational analysis (GRA) for the selection of sustainable building material suppliers. In the TOPSIS procedure, the objective weights based on intuitionistic fuzzy entropy were used instead of subjective weights directly obtained from decision-makers. Memari et al. [50] applied an intuitionistic fuzzy TOPSIS method to select the right sustainable supplier. Rouyendegh et al. [51] used an intuitionistic fuzzy TOPSIS to select the best green supplier for a company located in Ankara. The study was conducted with the help of 10 criteria, and three decision-makers select the best among four supplier companies. Dymova et al. [52] proposed the generalization of the intuitionistic fuzzy TOPSIS method in the framework of evidence theory.

This short literature review shows that several variants of the intuitionistic fuzzy TOPSIS approaches have been extensively investigated in the framework of decision-making problems. The main decisions to be made in the intuitionistic fuzzy TOPSIS-based approach are related to the ranking of alternatives and weights of criteria, construction reference points, and calculation distance measure. Table 1 summarizes the main differences in the TOPSIS methods based on an intuitionistic fuzzy framework.

## 4. The Intuitionistic Fuzzy TOPSIS-Based Model for Evaluating Socioeconomic Phenomena on the Basis of Survey Data

Let $O=\left\{O_{1},\;O_{2},\;\ldots ,\;O_{m}\right\}$ be the set of objects under survey evaluation, and let $C=\left\{C_{1},\;C_{2},\;\ldots ,\;C_{n}\right\}$ be the set of criteria for the objects are evaluated. We assume that respondents answered questions using different scales, which can be aggregated into three groups: “positive opinion about the object”, “negative opinion about the object”, and “no opinion or no answer”. As an example, let us take a question from the “Perception survey on the quality of life in European cities, 2019” (Table 2).

In this case, “positive opinions” would include answers 3 and 4. “Negative opinions” would include answers 1 and 2. The third group would include answers coded as 99. However, it should be noted that, in opinion research with the use of ordinal scales, reverse items are also used. An example of such an item is Q13 from the same survey (Table 3).

In such an inverse item, answers 1 and 2 would be considered “positive opinions”. The set of “negative opinions” would constitute answers 3 and 4. Answers coded as 99 would again form the third group.

The algorithm for the evaluation of socioeconomic phenomena, when survey data are represented by *IFSs*, using an IF-TOPSIS technique, is given in seven steps. Taking into consideration that the survey questions had the same importance in the evaluation of the objects, we assumed that the weights of criteria were equal. Let us recall that Maggino and Ruviglioni [53] noted that equal weights are used in most applications, whereby, “when the theoretical structure attributes to each indicator the same adequacy in defining the variable to be measured, the theoretical structure does not allow hypotheses to be consistently derived on differential weightings”.

The procedure is described below.

Step 1. Obtain performance data in the form of intuitionistic fuzzy values (*IFVs*) for *m* objects over *n* criteria.

In this step, the objects are evaluated according to criteria. The respondent’s opinions about object $O_{i}$ for each criterion $C_{j}$ are represented by *IFVs* $\left(\mu_{ij},\;\nu_{ij}\right)$, where $\mu_{ij}$ indicates the fraction of positive opinions about the *i*-th object with respect to the *j*-th criterion, $\nu_{ij}$ indicates the fraction of negative opinions about the *i*-th object with respect to the *j*-th criterion, and $\pi_{ij}$ represents the fraction of opinion type “do not know” or “no answer” for the *i*-th object with respect to the *j*-th criterion. Thus, we have $\pi_{ij}(x)=1-\mu_{ij}(x)-\nu_{ij}(x)$, i.e.,

(5) $$\mu_{ij}=\frac{p_{ij}}{N_{i}},\;\nu_{ij}=\frac{n_{ij}}{N_{i}},\;\pi_{ij}=\frac{h_{ij}}{N_{i}},$$

where $p_{ij}$ is the total number of respondents who positively evaluated the *i*-th object with respect to the *j*-th criterion, $n_{ij}$ is the total number of respondents who negatively evaluated the *i*-th object with respect to the *j*-th criterion, $h_{ij}$ is the total number of respondents with a hesitant opinion about the *i*-th object with respect to the *j*-th criterion, and $N_{i}$ is the total number of respondents who evaluated the *i*-th object. Let us note that $p_{ij}+n_{ij}+h_{ij}=N_{i}$. Clearly, instead of the total number of responses, we can use the percentage of relevant responses, which is common for secondary survey data.

Let us observe that the *i*-th object $O_{i}$ is represented by the following vector:

(6) $$O_{i}=[(\mu_{i1},\;\nu_{i1}),\;\ldots ,\;(\mu_{in},\;\nu_{in})],$$

where i=1, 2, …, m.

Step 2. Determine Intuitionistic Fuzzy Decision Matrix.

Subsequently, the intuitionistic fuzzy decision matrix is given in the following form:

(7) $$D=\left[\begin{array}{cccc} \left(\mu_{11},\;\nu_{11}\right) & \left(\mu_{12},\;\nu_{12}\right) & \ldots & \left(\mu_{1n},\;\nu_{1n}\right)\\ \left(\mu_{21},\;\nu_{21}\right) & \left(\mu_{22},\;\nu_{22}\right) & \ldots & \left(\mu_{2n},\;\nu_{2n}\right)\\ \ldots & \ldots & \ldots & \ldots \\ \left(\mu_{m1},\;\nu_{m1}\right) & \left(\mu_{m1},\;\nu_{m1}\right) & \ldots & \left(\mu_{mn},\;\nu_{mn}\right) \end{array}\right].$$

Step 3. Determine intuitionistic fuzzy positive-ideal and negative-ideal objects.

The intuitionistic fuzzy positive-ideal object is based on maximum *IFV* and has the following form:

(8) IFP=[(1, 0), …, (1, 0)].

The intuitionistic fuzzy negative-ideal object is based on minimum *IFV* and has the following form:

(9) IFN=[(0, 1), …, (0, 1)].

Step 4. Calculate the distance measures.

The distance measure from the fuzzy positive-ideal object $d^{+}(\;O_{i})=d(IFP,\;O_{i})$ and the distance measure from the fuzzy negative-ideal object $d^{-}(\;O_{i})=d(IFN,\;O_{i})$ are calculated using Equation (3).

Step 5. Calculate the intuitionistic fuzzy TOPSIS coefficient (*IFT).*

For each object, we calculated the ratio of the distance from the fuzzy negative-ideal object to the sum of the distance from the fuzzy negative-ideal object and fuzzy positive-ideal object. The intuitionistic fuzzy TOPSIS coefficient is defined as follows:

(10) $$IFT(O_{i})=\frac{d^{-}(\;O_{i})}{d^{-}(O_{i})+d^{+}(O_{i})}.$$

Step 6. Rank the objects by maximizing the coefficient $IFT(O_{i})$.

Let us observe that IFT∈[0, 1]. The highest value of $IFT(O_{i})$ is, thus, the highest position of the object $O_{i}$. Moreover, IFT=1 for IFP=[(1, 0), …, (1, 0)], and IFT=0 for IFN=[(0, 1), …, (0, 1)].

Step 7. Classify objects with respect to the level of socioeconomic phenomena.

Different classification criteria could be applied. We present two classification procedures that allowed distinguishing the objects according to the various levels of socioeconomic development with respect to the IF-TOPSIS procedure. The first is based on average and standard deviation measures, as described below.

Group 1: $IFT\in [\bar{IFT}+s(IFT);\;\max(IFT_{i})]$ represents a very high level of socioeconomic phenomena;
Group 2: $IFT\in [\bar{IFT};\;\;\bar{IFT}+s(IFT))$ represents a medium–high level of socioeconomic phenomena;
Group 3: $IFT\in [\bar{IFT}-s(IFT);\;\bar{IFT})$ represents a medium–low level of socioeconomic phenomena;
Group 4: $IFT\in [\min(IFT_{i});\;\bar{IFT}-s(IFT))$ represents a very low level of socioeconomic phenomena, where $\bar{IFT}$ and s(IFT) are the average value and the standard deviation of the coefficient.

The second procedure is based on the values of the coefficient IFT∈[0, 1], as described below.

Group 1: IFT∈[0.8, 1] represents a very high level of socioeconomic phenomena;
Group 2: IFT∈[0.6, 0.8) represents a high level of socioeconomic phenomena;
Group 3: IFT∈[0.4, 0.6) represents a medium level of socioeconomic phenomena;
Group 4: IFT∈[0.2, 0.4) represents a low level of socioeconomic phenomena;
Group 5: IFT∈[0, 0.2) represents a very low level of socioeconomic phenomena.

**Figure 1:**
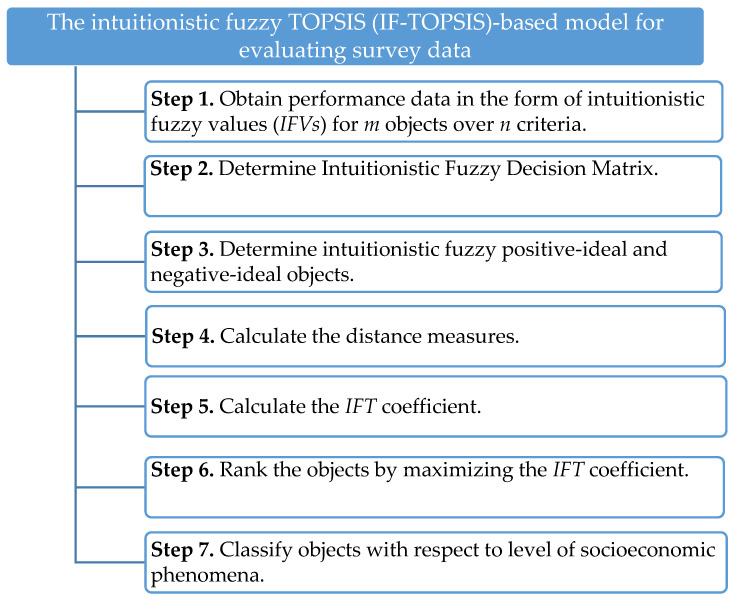
The IF-TOPSIS-based model for the evaluation of survey data.

The IF-TOPSIS-based model for the evaluation of questionnaires is presented in Figure 1.

## 5. Empirical Example

The proposed approach to the analysis of survey data, using the presented procedure and the IF-TOPSIS method, was used in the analysis of results from the fifth survey on quality of life in European cities. A growing interest in the quality of life category has been observed in scientific research conducted by representatives of various fields and disciplines of science, as well as in the practical dimension, i.e., in the daily life of individuals, groups, or entire communities, e.g., city inhabitants. A natural consequence of this interest is differences in views on the perception of the quality of life category and differences in the methodology of the conducted research, including the methods and tools used to measure it. Despite these differences, there is agreement on the fundamental assumptions of this category. Undoubtedly, quality of life should be treated as a complex, multidimensional construct. In this context, in the literature on the subject, one can find proposals for definitions of a global (holistic) nature, covering all aspects and dimensions of the quality of life, as well as of a partial (detailed or fragmentary) nature, which relate only to selected areas, e.g., health. Another important issue is taking into account the two dimensions of the quality of life category, objective and subjective, which is of particular importance for the practice of research in the field of measuring and assessing the quality of life. Following international recommendations and current research practice, this measurement should take into account both objective and subjective indicators. Both sets of indicators should be treated as complementary and give a complete picture of the quality of life in both its dimensions. Objective quality of life is sometimes equated with living conditions (standard of living) and includes all indicators of the nature of objective measures that allow the comparison of various parameters in the economic, social, or environmental dimensions that determine the life of individuals and societies. Subjective quality-of-life indicators take the form of value judgments that are formulated by an individual in relation to their own life in the context of previous experiences, desires, expectations, and system of values. It is worth emphasizing here that the relationships between quality of life in the objective and subjective dimensions are not clearly defined, i.e., the improvement of objective living conditions does not have to translate directly into an increase in the subjective sense of satisfaction with life. This is due to the fact that subjective quality of life is an individual matter, dependent on the unique perception of each person.

The fifth survey on quality of life in European cities was conducted for the European Commission by the IPSOS company. The survey covered the inhabitants of 83 cities in EU countries, EFTA countries, the UK, the Western Balkans, and Turkey. The study was conducted between 12 June and 27 September 2019, with a pause between 15 July and 1 September. A total of 700 interviews were completed in each city surveyed. This means that a total of 58,100 inhabitants of 83 cities participated in the study.

Our study aimed to measure and benchmark inhabitants’ satisfaction with life in selected cities using the IF-TOPSIS method. Inhabitants’ satisfaction, as a complex phenomenon, was characterized using 10 criteria: C_1_—satisfaction with public transport, C_2_—satisfaction with healthcare services, doctors, and hospitals, C_3_—satisfaction with sports facilities such as sports fields and indoor sports halls, C_4_—satisfaction with cultural facilities such as concert halls, theaters, museums, and libraries, C_5_—satisfaction with green spaces such as parks and gardens, C_6_—satisfaction with public spaces such as markets, squares, and pedestrian areas, C_7_—satisfaction with schools and other educational facilities, C_8_—satisfaction with the quality of the air, C_9_—satisfaction with the noise level, and C_10_—satisfaction with the cleanliness. In the assessment of criteria, a five-point measurement scale was used: very satisfied, rather satisfied, rather unsatisfied, very unsatisfied, and do not know/no answer.

The characteristics of the research sample in terms of sex, age, and level of education are presented in Table 4.

Due to the large number of cities covered by the survey, individual steps of the proposed procedure are presented for the example of three selected cities: London, Stockholm, and Vienna. The selected cities were among the top five most sustainable cities in 2018 according to the ARCADIS ranking. Edinburgh and Singapore, ranked third and fourth, respectively, were not included in the analyzed survey. The assessment of selected cities in terms of 10 criteria was presented in two manners by Eurostat. The first approach, using a four-point Likert scale, is presented in Table 5. The second approach was based on opinion aggregated into three classes: (1) unsatisfied, which included categories very unsatisfied and rather unsatisfied; (2) satisfied, which included categories rather satisfied and very satisfied; (3) respondents with answers of do not know/no answer/refused to answer (see Table 6).

These two ways of presenting data allow for two possibilities for the aggregation of data, as well as for the construction of two synthetic measures. The novel method IF-TOPSIS proposed in this paper was applied to the data, as presented in Table 6.

According to Equation (5), the respondents’ assessments were transformed into *IFVs* (Table 7).

Criterion assessments in the form of *IFVs* for all cities are listed in Table A1, Table A2 and Table A3 (Appendix A).

Assessments of cities in terms of the 10 criteria in the form of *IFVs* were used to construct an intuitionistic fuzzy decision matrix, a fragment of which is presented below for the described cities.

$$D=\begin{array}{c} \begin{array}{cccc} \;\;\;\;\;\;\;\;\;\;\;\;\;\;\;\;\;C_{1} & \;\;\;\;\;\;\;\;\;\;\;\;\;\;C_{2} & \;\;\;\;\;\;\;\ldots & \;\;\;\;\;\;\;C_{10} \end{array}\;\;\\ \begin{array}{c} \ldots \\ \mathrm{London}\\ \ldots \\ \mathrm{Stockholm}\\ \ldots \\ \mathrm{Wien}\\ \ldots \end{array}\left[\begin{array}{cccc} \ldots & \ldots & \ldots & \ldots \\ \left(0.710,\;0.159\right) & \left(0.806,\;0.153\right) & \ldots & \left(0.797,\;0.186\right)\\ \ldots & \ldots & \ldots & \\ (0.813,\;0.148 & (0.890,\;0.088 & \ldots & (0.630,\;0.356)\\ \ldots & \ldots & \ldots & \ldots \\ (0.962,\;0.033) & (0.919,\;0.063) & \ldots & (0.895,\;0.101)\\ \ldots & \ldots & \ldots & \ldots \end{array}\right] \end{array}.$$

We did not assign weights for individual criteria. According to the concept of sustainable development, all dimensions (social, environmental, and economic) and aspects (e.g., health, education, security) should be in balance, i.e., they are equally important for improving the quality of life, for both current and future generations. Recognizing that a given dimension or aspect of sustainable development has priority over others (has a higher weight) would be contrary to the idea of sustainable development.

The coordinates of intuitionistic fuzzy positive-ideal and negative-ideal objects were determined on the basis of the maximum and minimum *IFVs*, respectively (Table 8 and Table 9).

Using the normalized Euclidean distance in accordance with Equation (3), the distances of each city from the intuitionistic fuzzy positive-ideal and negative-ideal objects were calculated, and then the *IFT* coefficients were calculated (Table 10).

The values of IF-TOPSIS coefficients for all cities are presented in Table A4 (Appendix A).

The position of cities in the ranking was determined on the basis of the IF-TOPSIS coefficient values, in accordance with the principle that a higher value of the IF-TOPSIS coefficient denoted a higher position in the ranking (Table 11).

On the basis of the *IFT* coefficient values, the cities were classified into one of five classes according to the second procedure proposed in step 6 (Table 12).

**Figure 2:**
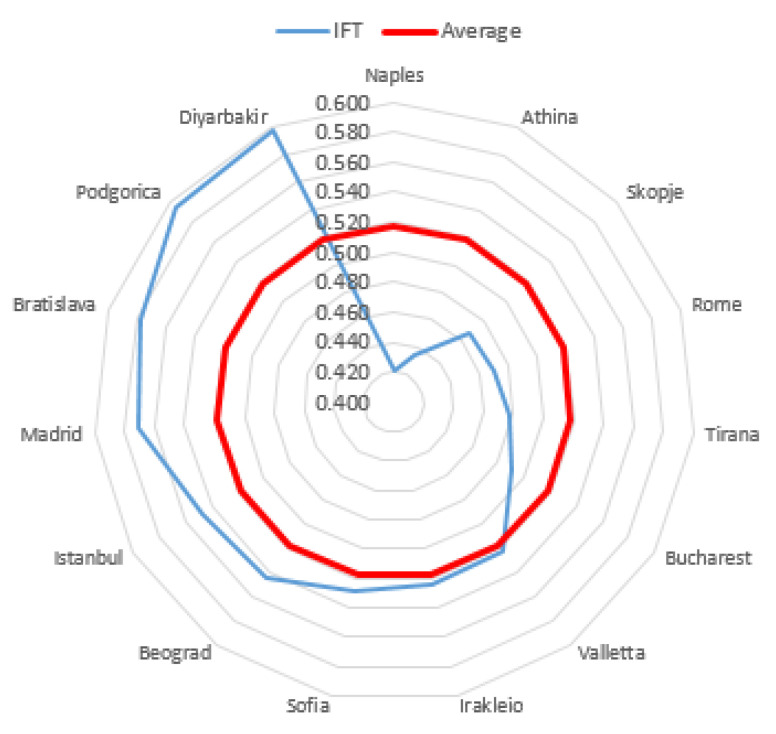
Cities with a medium level of quality of life of inhabitants compared with an average level of quality of life of inhabitants in this class.

**Figure 3:**
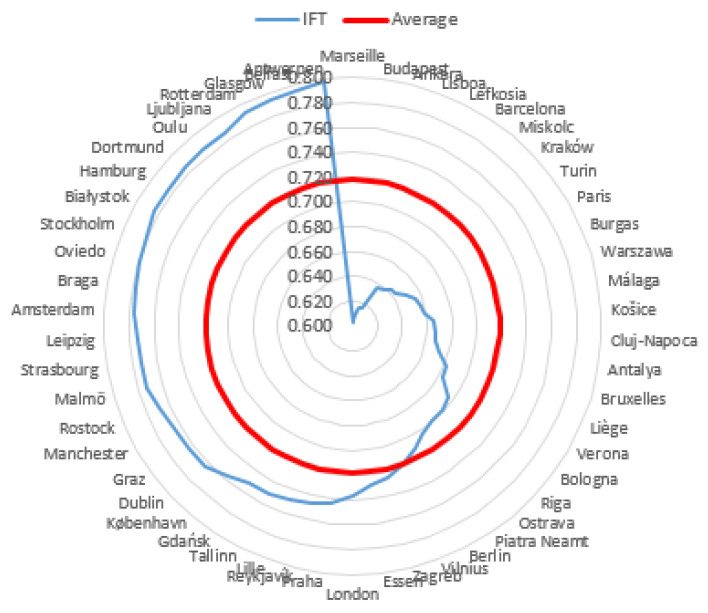
Cities with a high level of quality of life of inhabitants compared with an average level of quality of life of inhabitants in this class.

**Figure 4:**
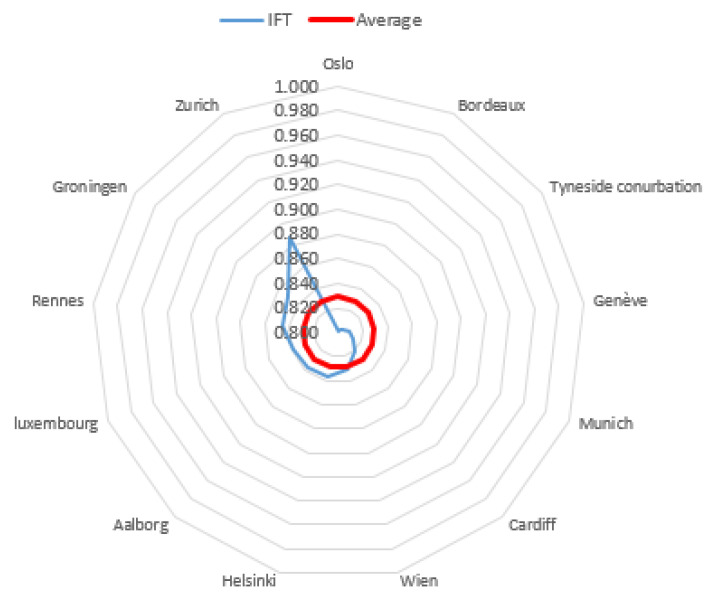
Cities with a very high level of quality of life of inhabitants compared with an average level of quality of life of inhabitants in this class.

The level of subjective quality of life in most cities was high (65.06% of the research sample). The highest level of this phenomenon was observed in 15.663% of cities. Zurich was the highest in the ranking. The value of the *IFT* coefficient for this city was 0.886. Palermo obtained the lowest rank with an *IFT* coefficient of 0.377. The inhabitants’ subjective quality of life of 18.072% of the cities was at the average level. Graphical representations of the obtained results, broken down into three city classes, distinguished on the basis of the inhabitants’ level of subjective quality of life, are presented in Figure 2, Figure 3 and Figure 4.

For the class with a low level of quality of life, it was not possible to prepare a chart because only Palermo was included in it.

Additionally, basic descriptive statistics were calculated for three classes (Table 13).

The most significant differentiation in terms of *IFT* coefficient values was observed in the group of cities with a medium level quality of life. Exactly six of the 15 cities in this group achieved *IFT* coefficient values below the group average (less than 0.518). The highest values were observed for the cities of Diyarbakir and Podgorica. These two cities were almost at the top of the medium class. The second group of cities, for which the subjective quality of life is high, was the most numerous with 54 cities.

Interestingly, the variation area, i.e., the difference between the highest and the lowest scores in this group, was almost equal to the class width and amounted to 0.195. At two opposite ends were Marseille with a score of 0.603 and Antwerpen with an *IFT* coefficient value of 0.798. Exactly 30 cities in this group were those for which the *IFT* coefficient value was equal to or higher than the group means. The most homogeneous and numerous group was the third group, which included cities with a very high quality of life. The best result was observed for Zurich with an *IFT* value of 0.886. Although this was the highest result in this group of cities, the obtained value was closer to the lower limit defining this class (*IFT* of 0.800).

Contrary to the medium and high groups, in the very high group, no city came close to the upper limit of this class, i.e., an *IFT* coefficient value equal to 1. However, it is worth emphasizing that cities with high and very high quality of life account for about 80% of all surveyed cities. The list also includes the one-element low class, featuring Palermo, and the empty very low class.

The results obtained for the cities with partial calculations presented significant discrepancies between sustainable development assessment based on objective indicators and quality of life assessment based on subjective indicators. London, the best city in the ARCADIS ranking, took 40th place in the ranking of inhabitants’ quality of life. Stockholm was ranked higher in the 23rd position. Vienna occupied the highest at the seventh position. It should be emphasized here that a high level of sustainable development of cities should lead to its inhabitants’ satisfaction. As shown by the results of this study, this relationship is not always so unambiguous. However, it should be emphasized that the discussed issue was not the goal of our analyses and would require much more in-depth research evaluating, e.g., the selection of indicators, the method of their measurement, the number of dimensions representing the discussed constructs, and aggregation methods.

Lastly, we compared the results obtained using the IF-TOPSIS method with the results obtained using the classical TOPSIS method (for the algorithm of classical TOPSIS, see, e.g., [5]). The use of the TOPSIS method required averaging the respondents’ ratings for each criterion. The numerical signs assigned to the four categories of the Likert scale in the Eurostat database were multiplied by the respective shares of the respondents’ answers. Thus, the ratings of the criteria fell within the range 1–4. In the classic TOPSIS variant, missing responses, refusals to answer, or “do not know” responses were not taken into account because they do not constitute a point on the Likert scale used in the study. The coordinates of the fuzzy positive-ideal and negative-ideal objects were assumed as 4 and 1. The positions of cities in the ranking obtained using the classic TOPSIS method are presented in Table 14.

The values of the coefficients for cities and the classification of cities in terms of subjective quality of life using the classic TOPSIS method are presented in Table A5 and Table A6 (Appendix A).

The values of the coefficients for cities calculated using the classic TOPSIS method decreased compared to the results obtained using the IF-TOPSIS method. The mean values of the coefficients using the IF-TOPSIS and TOPSIS methods were 0.695 and 0.578, respectively. Changes in the values of the coefficients influenced the classification of cities in terms of the subjective quality of life of their inhabitants. The class of cities with a low quality of life level grew, while none of the cities were classified into the group of cities with the highest quality of life level. The biggest changes took place in the class with the average level of quality of life, featuring a number of cities that came close to the high class.

The vast majority of cities changed their position in the ranking after applying the classic TOPSIS method. The Kendall tau correlation coefficient for the two compared rankings was Tau = 0.878 (*p*-value < 0.0001). In the case of 9.639% of cities, the use of these two methods did not affect the positions in the rankings. Most of the cities (18.072%) changed their position in the ranking by only one position. The analyzed group of cities also included such cities for which the choice of the method had a significant impact on the position in the ranking. These include Graz, Braga, and Dublin, for which position changes in the rankings amounted to 23, 15, and 14 places, respectively.

## 6. Conclusions and Discussions

The literature has proposed several tools for solving multicriteria decision-making problems in various real-life situations under different types of uncertainties (for example, see [55,56,57,58,59]). In this paper, we proposed an intuitionistic fuzzy TOPSIS (IF-TOPSIS) method to solve the problem of assessing socioeconomic phenomena on the basis of survey data. Firstly, the proposed approach does not require raw data, and it takes into consideration uncertainty in respondents’ opinions. This enables the evaluation of the phenomenon on the basis of aggregated secondary data by transforming these data into intuitionistic fuzzy values. The degree of membership to the intuitionistic fuzzy value is the fraction of positive opinions in all assessments of the object in terms of the selected criterion. The degree of non-membership to an intuitionistic fuzzy value is the fraction of negative opinions in all assessments of the object in terms of the selected criterion. The degree of uncertainty is estimated according to Definition 1. The degree of uncertainty is represented by the fraction of respondents who, for various reasons, did not express their opinion or chose the option “hard to say”, “I have no opinion”, etc.

Secondly, the proposed approach to the transformation of aggregate secondary data into the form of intuitionistic fuzzy values does not violate the assumptions related to the measurement level of ordinal scales, as well as permissible relations and transformations of their values. After data transformation, the evaluation of the complex phenomenon using the IF-TOPSIS method is based on arithmetic operations, comparisons, and transformations acceptable for intuitionistic fuzzy values.

The last distinguishing feature of the proposed approach is the method of determining the coordinates of the intuitionistic fuzzy positive-ideal and negative-ideal objects. In the case of complex phenomena assessed on the basis of continuous data, the coordinates of the intuitionistic fuzzy positive-ideal and negative-ideal objects are determined, respectively, on the basis of the maximum and minimum values observed in the set of objects. Researchers are usually unable to indicate the desired values of the criteria. This results, inter alia, to the fact that adding another object to the research sample may change the coordinates of the intuitionistic fuzzy positive-ideal and negative-ideal objects, thus changing the position of the objects in the ranking. The method of determining the coordinates proposed in the article uses the parameters of intuitionistic fuzzy values. Thus, in the case of an intuitionistic fuzzy positive-ideal object, the degrees of membership and non-membership to the intuitionistic fuzzy set are known and should be 1 and 0, respectively. The degrees of membership and non-membership to the intuitionistic fuzzy set for an intuitionistic fuzzy negative-ideal object will be 0 and 1, respectively. In this case, including the new object in the intuitionistic fuzzy decision matrix will have no effect on the coordinates of the intuitionistic fuzzy positive-ideal and negative-ideal objects. This approach is characterized by the additional advantage of comparability of the results over time because, in each time unit, the results of the object evaluation are always related to the same intuitionistic fuzzy positive-ideal and negative-ideal objects. Of course, it is also possible to use an approach based on the maximum and minimum values of the parameters of intuitionistic fuzzy values.

Comparing the IF-TOPSIS method with the classic TOPSIS method made it possible to indicate the strengths and weaknesses of the proposed approach in the analysis of survey data aggregated from public statistics. The proposed method is simple and does not require raw data. It is directly based on the data format provided by official statistics. The method does not differentiate the categories that make up the positive and negative opinions of the respondents, which results in the loss of a certain part of information. On the other hand, it takes into account an additional element, which is the uncertainty expressed in the lack of answers, refusal to answer, or ignorance of the answer, which is omitted in the classic variant of the TOPSIS method. In addition, the classic TOPSIS method requires a real number of criteria assessments. This necessitates aggregating the opinions of respondents using an arithmetic mean. Therefore, using the classic TOPSIS method, we are forced to assume the equality of intervals among the various categories that make up the measurement scale. This assumption is most often not met, which means that the evaluation of the criteria will be imprecise and may impact the final results obtained using the classic TOPSIS method.

The future research challenge is to propose a modification of the IF-TOPSIS method which will make it possible to take into account the distribution of ratings into individual categories on the side of positive and negative ratings. Future research will also focus on applying the proposed approach and the intuitionistic fuzzy TOPSIS method to analyze changes in the quality of life in cities in 2007–2020. The method of determining intuitionistic fuzzy positive-ideal and negative-ideal objects and the method of classifying cities in terms of the quality of life of their inhabitants proposed in the article will make it possible to identify cities with the smallest and greatest changes in quality of life. The proposed approach is also resistant to changes in the number of cities in particular editions of the survey. The analysis will also concern changes in individual classes in the period 2007–2020, because our study results indicated that there are cities located on the border of two classes, whereby it was difficult to classify them in terms of the quality of life of their inhabitants.

## Figures and Tables

**Table 1:** A classification of TOPSIS methods based on an intuitionistic fuzzy framework with respect to rating the alternatives, rating the weights of criteria, reference points, and distance measure.

Authors	Rating the Alternatives	Rating the Weights of Criteria	Reference Points	Distance Measure
Joshi & Kumar [45]	Intuitionistic fuzzy numbers	Intuitionistic fuzzy entropy	Max and min	Max distance based on two parameters
Büyüközkan and Güleryüz [46] Boran et al. [43] Zulqarnain and Dayan [47] Memari et al. [50] Rouyendegh et al. [51]	Linguistic terms expressed by intuitionistic fuzzy sets	Linguistic terms expressed by intuitionistic fuzzy sets	Max and min	Euclidean distance based on three parameters
Shen et al. [30]	Intuitionistic fuzzy numbers	Maximizing deviation method	Max and min	Authors distance measure between the two intuitionistic fuzzy numbers
Rouyendegh [32]	Linguistic terms expressed by intuitionistic fuzzy sets	ANP	Max and min	Hamming distance
Rouyendegh et al. [48]	Intuitionistic fuzzy numbers	Intuitionistic fuzzy numbers	Max and min	Euclidean distance based on three parameters
Chen [49]	Intuitionistic fuzzy numbers	Intuitionistic fuzzy entropy	Max and min	Euclidean distance based on three parameters

**Table 2:** Example of scale item.

Item	Strongly Disagree	Somewhat Disagree	Somewhat Agree	Strongly Agree	Do Not Know/ No Answer/ Refused to Answer
The procedures used by my local public administration are straightforward and easy to understand	1	2	3	4	99

**Table 3:** Example of reverse scale item.

Item	Strongly Disagree	Somewhat Disagree	Somewhat Agree	Strongly Agree	Do Not Know/ No Answer/ Refused to Answer
There is corruption in my local administration	1	2	3	4	99

**Table 4:** Sociodemographic characteristic of respondents.

Feature	Category	Percentage
Sex	Male	47.739%
	Female	52.261%
Age	15–19	4.965%
	20–24	9.288%
	25–34	18.683%
	35–44	17.592%
	45–54	15.872%
	55–64	13.920%
	65–74	11.407%
	75+	8.273%
Education	Less than primary education	0.173%
	Primary education	1.308%
	Lower secondary education	10.389%
	Upper secondary education	35.257%
	Postsecondary nontertiary education	8.056%
	Short-cycle tertiary education	12.886%
	Bachelor or equivalent	18.269%
	Master or equivalent	10.986%
	Doctoral or equivalent	2.221%
	Do not know/no answer/refused to answer	0.455%

Source: [54].

**Table 5:** Assessment of cities (four-point Likert scale).

City	Category *	C_1_	C_2_	C_3_	C_4_	C_5_	C_6_	C_7_	C_8_	C_9_	C_10_
	1	2.475%	8.483%	6.754%	4.651%	1.630%	3.856%	7.673%	12.667%	6.632%	9.070%
	2	11.307%	18.444%	13.397%	12.601%	5.811%	9.753%	9.008%	31.283%	17.195%	24.155%
London	3	41.520%	40.197%	42.621%	33.230%	36.469%	54.561%	34.770%	39.145%	48.834%	49.034%
	4	42.453%	29.712%	23.608%	45.340%	55.660%	28.366%	26.621%	14.478%	25.731%	16.860%
	99	2.244%	3.163%	13.620%	4.179%	0.430%	3.464%	21.928%	2.426%	1.608%	0.882%
	1	2.588%	2.622%	9.875%	3.982%	1.320%	1.990%	2.457%	5.883%	5.449%	6.919%
	2	13.835%	18.048%	11.525%	5.887%	8.025%	9.158%	10.767%	14.454%	16.355%	21.308%
Stockholm	3	46.559%	48.850%	38.630%	34.112%	40.919%	58.368%	44.559%	51.299%	52.320%	51.024%
	4	33.821%	29.670%	19.117%	54.446%	49.637%	27.668%	21.765%	26.342%	24.438%	19.811%
	99	3.198%	0.811%	20.853%	1.572%	0.098%	2.817%	20.452%	2.022%	1.438%	0.938%
	1	1.268%	3.553%	5.429%	1.639%	2.050%	1.663%	3.128%	3.052%	5.765%	1.473%
	2	3.309%	10.632%	12.052%	2.864%	7.974%	8.575%	10.848%	11.917%	17.973%	11.517%
Wien	3	25.015%	37.529%	40.362%	18.672%	31.101%	47.463%	40.533%	47.846%	48.910%	48.793%
	4	69.961%	47.232%	23.871%	75.747%	58.358%	39.779%	31.573%	36.537%	25.648%	37.886%
	99	0.446%	1.054%	18.287%	1.077%	0.516%	2.519%	13.919%	0.648%	1.704%	0.331%
	Total	700	700	700	700	700	700	700	700	700	700

* 1—very unsatisfied, 2—rather unsatisfied, 3—rather satisfied, 4—very satisfied, 99—do not know/no answer/refused to answer. Source: [54].

**Table 6:** Assessment of cities (classes).

City	Classes *	C_1_	C_2_	C_3_	C_4_	C_5_	C_6_	C_7_	C_8_	C_9_	C_10_
	Unsatisfied	13.783%	26.928%	20.151%	17.252%	7.441%	13.609%	16.681%	43.951%	23.827%	33.225%
London	Satisfied	83.973%	69.909%	66.229%	78.570%	92.129%	82.927%	61.391%	53.623%	74.565%	65.894%
	99	2.244%	3.163%	13.620%	4.179%	0.430%	3.464%	21.928%	2.426%	1.608%	0.882%
	Unsatisfied	16.422%	20.669%	21.400%	9.870%	9.346%	11.147%	13.224%	20.337%	21.804%	28.227%
Stockholm	Satisfied	80.380%	78.520%	57.747%	88.558%	90.556%	86.036%	66.324%	77.641%	76.758%	70.835%
	99	3.198%	0.811%	20.853%	1.572%	0.098%	2.817%	20.452%	2.022%	1.438%	0.938%
	Unsatisfied	4.578%	14.185%	17.481%	4.504%	10.025%	10.239%	13.975%	14.969%	23.738%	12.990%
Wien	Satisfied	94.976%	84.761%	64.233%	94.419%	89.459%	87.242%	72.106%	84.383%	74.558%	86.679%
	99	0.446%	1.054%	18.287%	1.077%	0.516%	2.519%	13.919%	0.648%	1.704%	0.331%
	Total	700	700	700	700	700	700	700	700	700	700

* Unsatisfied (1—very unsatisfied, 2—rather unsatisfied), satisfied (3—rather satisfied, 4—very satisfied), 99—do not know/no answer/refused to answer. Source: [54].

**Table 7:** Assessment of cities with the use of *IFVs.*

City	Parameter	C_1_	C_2_	C_3_	C_4_	C_5_	C_6_	C_7_	C_8_	C_9_	C_10_
London	ν	0.138	0.269	0.202	0.173	0.074	0.136	0.167	0.440	0.238	0.332
	μ	0.840	0.699	0.662	0.786	0.921	0.829	0.614	0.536	0.746	0.659
	π	0.022	0.032	0.136	0.042	0.004	0.035	0.219	0.024	0.016	0.009
Stockholm	ν	0.164	0.207	0.214	0.099	0.093	0.111	0.132	0.203	0.218	0.282
	μ	0.804	0.785	0.577	0.886	0.906	0.860	0.663	0.776	0.768	0.708
	π	0.032	0.008	0.209	0.016	0.001	0.028	0.205	0.020	0.014	0.009
Wien	ν	0.046	0.142	0.175	0.045	0.100	0.102	0.140	0.150	0.237	0.130
	μ	0.950	0.848	0.642	0.944	0.895	0.872	0.721	0.844	0.746	0.867
	π	0.004	0.011	0.183	0.011	0.005	0.025	0.139	0.006	0.017	0.003

**Table 8:** The coordinates of intuitionistic fuzzy positive-ideal objects.

Parameter	C_1_	C_2_	C_3_	C_4_	C_5_	C_6_	C_7_	C_8_	C_9_	C_10_
ν	0	0	0	0	0	0	0	0	0	0
μ	1	1	1	1	1	1	1	1	1	1
π	0	0	0	0	0	0	0	0	0	0

**Table 9:** The coordinates of intuitionistic fuzzy negative-ideal objects.

Parameter	C_1_	C_2_	C_3_	C_4_	C_5_	C_6_	C_7_	C_8_	C_9_	C_10_
ν	1	1	1	1	1	1	1	1	1	1
μ	0	0	0	0	0	0	0	0	0	0
π	0	0	0	0	0	0	0	0	0	0

**Table 10:** Distances and *IFT* values.

City	Distance from *IFP*	Distance from *IFN*	*IFT* Coefficient
London	0.274	0.766	0.737
Stockholm	0.227	0.808	0.781
Wien	0.175	0.858	0.831

**Table 11:** Ranking of cities in terms of quality of life based on IF-TOPSIS method.

City	Rank	City	Rank	City	Rank	City	Rank
Aalborg	5	Diyarbakir	68	Ljubljana	18	Reykjavík	38
Amsterdam	26	Dortmund	20	London	40	Riga	47
Ankara	65	Dublin	33	Luxembourg	4	Rome	79
Antalya	52	Essen	41	Madrid	71	Rostock	30
Antwerpen	14	Gdańsk	35	Málaga	55	Rotterdam	17
Athina	81	Genève	10	Malmö	29	Skopje	80
Barcelona	62	Glasgow	16	Manchester	31	Sofia	74
Belfast	15	Graz	32	Marseille	67	Stockholm	23
Beograd	73	Groningen	2	Miskolc	61	Strasbourg	28
Berlin	44	Hamburg	21	Munich	9	Tallinn	36
Bialystok	22	Helsinki	6	Naples	82	Tirana	78
Bologna	48	Irakleio	75	Oslo	13	Turin	59
Bordeaux	12	Istanbul	72	Ostrava	46	Tyneside conurbation	11
Braga	25	København	34	Oulu	19	Valletta	76
Bratislava	70	Košice	54	Oviedo	24	Verona	49
Bruxelles	51	Kraków	60	Palermo	83	Vilnius	43
Bucharest	77	Lefkosia	63	Paris	58	Warszawa	56
Budapest	66	Leipzig	27	Piatra Neamt	45	Wien	7
Burgas	57	Liège	50	Podgorica	69	Zagreb	42
Cardiff	8	Lille	37	Praha	39	Zurich	1
Cluj-Napoca	53	Lisboa	64	Rennes	3	-	-

**Table 12:** Classification of cities with respect to level of quality of life.

City	Level	City	Level	City	Level	City	Level
Aalborg	very high	Diyarbakir	medium	Ljubljana	high	Reykjavík	high
Amsterdam	high	Dortmund	high	London	high	Riga	high
Ankara	high	Dublin	high	Luxembourg	very high	Rome	medium
Antalya	high	Essen	high	Madrid	medium	Rostock	high
Antwerpen	high	Gdańsk	high	Málaga	high	Rotterdam	high
Athina	medium	Genève	very high	Malmö	high	Skopje	medium
Barcelona	high	Glasgow	high	Manchester	high	Sofia	medium
Belfast	high	Graz	high	Marseille	high	Stockholm	high
Beograd	medium	Groningen	very high	Miskolc	high	Strasbourg	high
Berlin	high	Hamburg	high	Munich	very high	Tallinn	high
Białystok	high	Helsinki	very high	Naples	medium	Tirana	medium
Bologna	high	Irakleio	medium	Oslo	very high	Turin	high
Bordeaux	very high	Istanbul	medium	Ostrava	high	Tyneside conurbation	very high
Braga	high	København	high	Oulu	high	Valletta	medium
Bratislava	medium	Košice	high	Oviedo	high	Verona	high
Bruxelles	high	Kraków	high	Palermo	low	Vilnius	high
Bucharest	medium	Lefkosia	high	Paris	high	Warszawa	high
Budapest	high	Leipzig	high	Piatra Neamt	high	Wien	very high
Burgas	high	Liège	high	Podgorica	medium	Zagreb	high
Cardiff	very high	Lille	high	Praha	high	Zurich	very high
Cluj-Napoca	high	Lisboa	high	Rennes	very high	-	-

**Table 13:** Descriptive statistics for classes describing the quality of life in cities.

Descriptive Statistics	Level of Quality of Life		
	Medium	High	Very High
Min	0.421	0.603	0.800
Max	0.598	0.798	0.886
Average	0.518	0.718	0.829
Standard deviation	0.056	0.061	0.024
Coefficient of variation	0.109	0.085	0.029

**Table 14:** Ranking of cities in terms of quality of life based on TOPSIS method.

City	Rank	City	Rank	City	Rank	City	Rank
Aalborg	4	Diyarbakir	62	Ljubljana	13	Reykjavík	43
Amsterdam	25	Dortmund	24	London	35	Riga	55
Ankara	64	Dublin	19	Luxembourg	6	Rome	75
Antalya	46	Essen	41	Madrid	70	Rostock	34
Antwerpen	17	Gdańsk	37	Málaga	48	Rotterdam	20
Athina	82	Genève	15	Malmö	30	Skopje	78
Barcelona	60	Glasgow	12	Manchester	28	Sofia	76
Belfast	11	Graz	9	Marseille	65	Stockholm	26
Beograd	73	Groningen	3	Miskolc	66	Strasbourg	21
Berlin	42	Hamburg	23	Munich	8	Tallinn	44
Bialystok	22	Helsinki	10	Naples	80	Tirana	81
Bologna	45	Irakleio	74	Oslo	18	Turin	59
Bordeaux	14	Istanbul	72	Ostrava	39	Tyneside conurbation	16
Braga	40	København	33	Oulu	27	Valletta	77
Bratislava	71	Košice	50	Oviedo	32	Verona	47
Bruxelles	56	Kraków	58	Palermo	83	Vilnius	52
Bucharest	79	Lefkosia	67	Paris	49	Warszawa	61
Budapest	69	Leipzig	31	Piatra Neamt	51	Wien	2
Burgas	57	Liège	53	Podgorica	63	Zagreb	38
Cardiff	7	Lille	36	Praha	29	Zurich	1
Cluj-Napoca	54	Lisboa	68	Rennes	5	-	-

## Data Availability

Not applicable (for secondary data analysis, see [54]).

## Appendix A

**Table A1:** Degrees of non-membership to *IFVs* for cities.

City	C_1_	C_2_	C_3_	C_4_	C_5_	C_6_	C_7_	C_8_	C_9_	C_10_
Aalborg	0.159	0.153	0.084	0.045	0.075	0.125	0.071	0.114	0.142	0.186
Amsterdam	0.148	0.088	0.107	0.061	0.114	0.121	0.105	0.286	0.317	0.356
Ankara	0.383	0.361	0.349	0.334	0.300	0.347	0.404	0.355	0.455	0.367
Antalya	0.360	0.325	0.277	0.277	0.229	0.280	0.340	0.266	0.394	0.251
Antwerpen	0.252	0.076	0.097	0.077	0.148	0.114	0.100	0.349	0.252	0.217
Athina	0.253	0.627	0.535	0.323	0.707	0.647	0.366	0.708	0.667	0.697
Barcelona	0.226	0.315	0.248	0.189	0.294	0.181	0.276	0.624	0.563	0.386
Belfast	0.218	0.236	0.183	0.085	0.150	0.171	0.106	0.156	0.147	0.262
Beograd	0.544	0.584	0.222	0.204	0.370	0.374	0.339	0.582	0.500	0.626
Berlin	0.135	0.155	0.235	0.117	0.126	0.200	0.282	0.278	0.397	0.507
Białystok	0.143	0.509	0.132	0.089	0.086	0.100	0.131	0.122	0.198	0.075
Bologna	0.271	0.178	0.136	0.108	0.135	0.167	0.150	0.535	0.402	0.494
Bordeaux	0.143	0.107	0.160	0.145	0.146	0.111	0.109	0.219	0.254	0.326
Braga	0.209	0.239	0.186	0.208	0.273	0.121	0.094	0.168	0.246	0.178
Bratislava	0.309	0.522	0.412	0.131	0.444	0.290	0.197	0.436	0.393	0.679
Bruxelles	0.260	0.142	0.195	0.115	0.150	0.194	0.270	0.486	0.451	0.583
Bucharest	0.420	0.537	0.348	0.179	0.362	0.437	0.391	0.794	0.684	0.617
Budapest	0.254	0.570	0.221	0.105	0.295	0.198	0.263	0.508	0.495	0.568
Burgas	0.111	0.539	0.176	0.193	0.171	0.167	0.135	0.685	0.443	0.281
Cardiff	0.189	0.177	0.133	0.046	0.080	0.121	0.070	0.144	0.200	0.247
Cluj-Napoca	0.159	0.434	0.146	0.103	0.312	0.253	0.219	0.480	0.501	0.305
Diyarbakir	0.432	0.408	0.434	0.364	0.307	0.264	0.448	0.261	0.454	0.438
Dortmund	0.109	0.162	0.170	0.076	0.090	0.157	0.130	0.196	0.245	0.369
Dublin	0.256	0.384	0.193	0.117	0.129	0.235	0.134	0.153	0.139	0.367
Essen	0.248	0.122	0.333	0.133	0.156	0.213	0.192	0.201	0.280	0.408
Gdańsk	0.123	0.488	0.116	0.070	0.153	0.150	0.110	0.219	0.326	0.238
Genève	0.135	0.085	0.090	0.093	0.063	0.096	0.072	0.284	0.310	0.240
Glasgow	0.178	0.170	0.144	0.077	0.107	0.159	0.126	0.241	0.147	0.347
Graz	0.228	0.082	0.177	0.114	0.220	0.134	0.085	0.501	0.240	0.180
Groningen	0.153	0.067	0.088	0.055	0.092	0.081	0.043	0.150	0.190	0.174
Hamburg	0.101	0.139	0.186	0.073	0.103	0.164	0.179	0.198	0.284	0.272
Helsinki	0.098	0.233	0.093	0.036	0.062	0.151	0.075	0.113	0.173	0.186
Irakleio	0.314	0.510	0.378	0.424	0.684	0.484	0.336	0.258	0.526	0.614
Istanbul	0.382	0.371	0.445	0.332	0.432	0.433	0.469	0.460	0.648	0.369
København	0.183	0.159	0.197	0.093	0.083	0.147	0.151	0.299	0.298	0.283
Košice	0.404	0.382	0.346	0.108	0.265	0.134	0.124	0.431	0.414	0.363
Kraków	0.192	0.479	0.146	0.087	0.264	0.146	0.116	0.807	0.504	0.321
Lefkosia	0.381	0.349	0.320	0.319	0.449	0.426	0.118	0.336	0.348	0.369
Leipzig	0.185	0.159	0.160	0.070	0.090	0.121	0.185	0.149	0.289	0.280
Liège	0.370	0.116	0.187	0.154	0.235	0.225	0.137	0.429	0.299	0.577
Lille	0.174	0.109	0.122	0.110	0.171	0.188	0.114	0.423	0.332	0.393
Lisboa	0.367	0.361	0.282	0.225	0.293	0.262	0.189	0.433	0.479	0.567
Ljubljana	0.226	0.296	0.152	0.119	0.131	0.146	0.115	0.243	0.302	0.142
London	0.138	0.269	0.202	0.173	0.074	0.136	0.167	0.440	0.238	0.332
Luxembourg	0.158	0.132	0.120	0.087	0.103	0.097	0.131	0.183	0.220	0.063
Madrid	0.241	0.340	0.376	0.314	0.318	0.280	0.370	0.654	0.514	0.616
Málaga	0.189	0.338	0.283	0.201	0.366	0.232	0.205	0.271	0.475	0.575
Malmö	0.213	0.301	0.106	0.062	0.041	0.098	0.232	0.172	0.133	0.311
Manchester	0.119	0.188	0.174	0.151	0.192	0.170	0.120	0.235	0.169	0.393
Marseille	0.282	0.157	0.341	0.212	0.343	0.390	0.238	0.507	0.431	0.742
Miskolc	0.254	0.573	0.237	0.087	0.253	0.200	0.160	0.460	0.320	0.511
Munich	0.148	0.080	0.123	0.066	0.057	0.120	0.152	0.206	0.257	0.125
Naples	0.664	0.546	0.608	0.363	0.687	0.503	0.380	0.655	0.575	0.743
Oslo	0.105	0.105	0.226	0.082	0.065	0.173	0.084	0.231	0.231	0.289
Ostrava	0.102	0.149	0.135	0.088	0.101	0.230	0.106	0.739	0.358	0.393
Oulu	0.431	0.332	0.111	0.053	0.095	0.152	0.061	0.108	0.118	0.143
Oviedo	0.217	0.205	0.223	0.196	0.165	0.097	0.173	0.223	0.259	0.070
Palermo	0.744	0.608	0.615	0.354	0.637	0.532	0.450	0.633	0.673	0.913
Paris	0.233	0.196	0.290	0.113	0.155	0.181	0.152	0.694	0.490	0.572
Piatra Neamt	0.198	0.514	0.212	0.229	0.161	0.345	0.177	0.159	0.228	0.172
Podgorica	0.575	0.512	0.349	0.385	0.445	0.258	0.271	0.404	0.331	0.386
Praha	0.096	0.142	0.122	0.087	0.206	0.169	0.098	0.383	0.428	0.421
Rennes	0.144	0.136	0.110	0.097	0.066	0.101	0.076	0.203	0.182	0.207
Reykjavík	0.260	0.273	0.091	0.072	0.176	0.196	0.126	0.172	0.200	0.272
Riga	0.195	0.504	0.256	0.106	0.130	0.214	0.231	0.299	0.264	0.254
Rome	0.719	0.490	0.353	0.289	0.402	0.460	0.373	0.676	0.526	0.911
Rostock	0.087	0.134	0.162	0.293	0.153	0.145	0.121	0.154	0.153	0.273
Rotterdam	0.073	0.093	0.119	0.076	0.159	0.074	0.089	0.362	0.226	0.320
Skopje	0.344	0.642	0.321	0.317	0.558	0.490	0.329	0.875	0.580	0.786
Sofia	0.188	0.494	0.362	0.195	0.341	0.386	0.317	0.704	0.609	0.654
Stockholm	0.164	0.207	0.214	0.099	0.093	0.111	0.132	0.203	0.218	0.282
Strasbourg	0.127	0.128	0.145	0.064	0.106	0.110	0.112	0.486	0.323	0.255
Tallinn	0.133	0.388	0.135	0.076	0.122	0.175	0.102	0.216	0.289	0.202
Tirana	0.671	0.545	0.543	0.451	0.533	0.413	0.278	0.720	0.549	0.470
Turin	0.345	0.272	0.179	0.100	0.186	0.145	0.202	0.669	0.440	0.542
Tyneside conurbation	0.113	0.139	0.158	0.136	0.144	0.152	0.078	0.142	0.146	0.310
Valletta	0.301	0.224	0.353	0.514	0.554	0.535	0.111	0.648	0.544	0.455
Verona	0.319	0.171	0.238	0.157	0.258	0.245	0.134	0.534	0.421	0.308
Vilnius	0.194	0.373	0.260	0.088	0.125	0.160	0.228	0.263	0.244	0.212
Warszawa	0.147	0.568	0.147	0.071	0.154	0.249	0.154	0.494	0.521	0.325
Wien	0.046	0.142	0.175	0.045	0.100	0.102	0.140	0.150	0.237	0.130
Zagreb	0.206	0.339	0.344	0.101	0.182	0.176	0.244	0.293	0.328	0.294
Zurich	0.033	0.063	0.052	0.032	0.093	0.106	0.054	0.072	0.201	0.101

**Table A2:** Degrees of membership to *IFVs* for cities.

City	C_1_	C_2_	C_3_	C_4_	C_5_	C_6_	C_7_	C_8_	C_9_	C_10_
Aalborg	0.710	0.806	0.758	0.917	0.902	0.819	0.829	0.860	0.847	0.797
Amsterdam	0.813	0.890	0.755	0.868	0.876	0.864	0.685	0.664	0.663	0.630
Ankara	0.560	0.638	0.484	0.539	0.687	0.644	0.523	0.637	0.541	0.630
Antalya	0.538	0.667	0.566	0.614	0.764	0.705	0.553	0.724	0.591	0.744
Antwerpen	0.695	0.917	0.807	0.894	0.842	0.881	0.849	0.631	0.722	0.759
Athina	0.705	0.339	0.369	0.638	0.289	0.344	0.489	0.279	0.323	0.301
Barcelona	0.724	0.672	0.657	0.744	0.697	0.798	0.599	0.364	0.433	0.598
Belfast	0.720	0.739	0.670	0.874	0.844	0.803	0.803	0.823	0.825	0.730
Beograd	0.365	0.398	0.707	0.754	0.626	0.610	0.608	0.400	0.486	0.366
Berlin	0.829	0.831	0.587	0.841	0.868	0.797	0.544	0.701	0.596	0.480
Białystok	0.739	0.455	0.758	0.875	0.909	0.892	0.734	0.866	0.797	0.915
Bologna	0.672	0.798	0.712	0.834	0.853	0.816	0.705	0.454	0.585	0.502
Bordeaux	0.834	0.885	0.762	0.821	0.838	0.876	0.808	0.767	0.740	0.664
Braga	0.640	0.749	0.728	0.732	0.715	0.868	0.853	0.817	0.751	0.818
Bratislava	0.570	0.449	0.466	0.786	0.544	0.672	0.607	0.526	0.596	0.310
Bruxelles	0.698	0.843	0.665	0.815	0.840	0.784	0.603	0.504	0.539	0.409
Bucharest	0.482	0.419	0.432	0.689	0.615	0.547	0.481	0.197	0.307	0.369
Budapest	0.681	0.400	0.550	0.797	0.693	0.789	0.503	0.487	0.491	0.429
Burgas	0.782	0.432	0.680	0.697	0.811	0.820	0.703	0.296	0.543	0.711
Cardiff	0.735	0.808	0.777	0.925	0.908	0.863	0.756	0.837	0.785	0.739
Cluj-Napoca	0.690	0.521	0.728	0.831	0.660	0.732	0.695	0.506	0.485	0.687
Diyarbakir	0.520	0.582	0.443	0.554	0.692	0.722	0.538	0.708	0.542	0.544
Dortmund	0.832	0.807	0.683	0.881	0.907	0.820	0.726	0.786	0.721	0.615
Dublin	0.720	0.603	0.745	0.873	0.865	0.745	0.796	0.834	0.856	0.624
Essen	0.660	0.863	0.523	0.823	0.830	0.773	0.644	0.775	0.702	0.586
Gdańsk	0.811	0.481	0.769	0.882	0.835	0.835	0.714	0.759	0.657	0.739
Genève	0.822	0.893	0.780	0.838	0.918	0.891	0.814	0.692	0.667	0.742
Glasgow	0.768	0.808	0.784	0.882	0.879	0.810	0.707	0.737	0.842	0.642
Graz	0.757	0.904	0.667	0.861	0.768	0.855	0.836	0.489	0.753	0.814
Groningen	0.757	0.904	0.767	0.892	0.898	0.890	0.859	0.819	0.806	0.812
Hamburg	0.878	0.825	0.634	0.900	0.889	0.824	0.643	0.782	0.690	0.719
Helsinki	0.870	0.737	0.816	0.845	0.918	0.831	0.764	0.878	0.807	0.797
Irakleio	0.559	0.478	0.534	0.526	0.308	0.499	0.558	0.736	0.474	0.368
Istanbul	0.569	0.628	0.461	0.567	0.558	0.560	0.497	0.527	0.344	0.618
København	0.776	0.789	0.587	0.842	0.900	0.803	0.652	0.661	0.687	0.708
Košice	0.521	0.594	0.554	0.833	0.727	0.832	0.746	0.537	0.564	0.627
Kraków	0.766	0.503	0.732	0.892	0.716	0.839	0.712	0.179	0.480	0.675
Lefkosia	0.400	0.612	0.496	0.590	0.536	0.558	0.787	0.624	0.647	0.622
Leipzig	0.774	0.831	0.630	0.907	0.900	0.856	0.555	0.828	0.685	0.707
Liège	0.563	0.875	0.680	0.779	0.735	0.738	0.764	0.535	0.663	0.410
Lille	0.766	0.881	0.805	0.812	0.807	0.786	0.821	0.546	0.656	0.596
Lisboa	0.544	0.604	0.565	0.678	0.670	0.722	0.676	0.520	0.513	0.416
Ljubljana	0.721	0.684	0.749	0.851	0.868	0.841	0.802	0.743	0.694	0.855
London	0.840	0.699	0.662	0.786	0.921	0.829	0.614	0.536	0.746	0.659
Luxembourg	0.813	0.851	0.790	0.844	0.884	0.876	0.747	0.801	0.766	0.937
Madrid	0.724	0.632	0.527	0.619	0.674	0.697	0.512	0.319	0.464	0.383
Málaga	0.744	0.648	0.663	0.751	0.608	0.745	0.698	0.701	0.519	0.422
Malmö	0.723	0.663	0.705	0.908	0.953	0.886	0.523	0.818	0.849	0.679
Manchester	0.813	0.788	0.698	0.795	0.786	0.808	0.685	0.739	0.809	0.588
Marseille	0.667	0.831	0.540	0.743	0.635	0.593	0.633	0.472	0.565	0.253
Miskolc	0.630	0.405	0.551	0.753	0.732	0.762	0.626	0.536	0.674	0.481
Munich	0.837	0.896	0.691	0.905	0.931	0.858	0.628	0.778	0.716	0.865
Naples	0.297	0.441	0.340	0.611	0.298	0.481	0.570	0.333	0.416	0.253
Oslo	0.881	0.869	0.651	0.888	0.929	0.798	0.743	0.754	0.755	0.702
Ostrava	0.818	0.804	0.753	0.872	0.885	0.736	0.796	0.250	0.623	0.597
Oulu	0.476	0.633	0.835	0.861	0.878	0.825	0.837	0.883	0.859	0.848
Oviedo	0.701	0.769	0.679	0.729	0.825	0.898	0.663	0.750	0.735	0.921
Palermo	0.207	0.366	0.286	0.598	0.341	0.464	0.474	0.349	0.320	0.076
Paris	0.749	0.795	0.600	0.857	0.836	0.805	0.690	0.293	0.502	0.428
Piatra Neamt	0.596	0.433	0.615	0.651	0.804	0.623	0.707	0.835	0.760	0.817
Podgorica	0.317	0.479	0.638	0.594	0.553	0.734	0.712	0.592	0.664	0.611
Praha	0.874	0.852	0.781	0.861	0.786	0.811	0.791	0.598	0.561	0.569
Rennes	0.835	0.837	0.817	0.870	0.919	0.882	0.862	0.781	0.807	0.787
Reykjavík	0.427	0.660	0.812	0.871	0.752	0.746	0.759	0.788	0.736	0.712
Riga	0.693	0.446	0.530	0.840	0.847	0.752	0.572	0.682	0.720	0.738
Rome	0.253	0.496	0.558	0.688	0.588	0.534	0.535	0.315	0.463	0.082
Rostock	0.855	0.840	0.643	0.620	0.838	0.836	0.550	0.839	0.841	0.722
Rotterdam	0.889	0.862	0.755	0.855	0.829	0.910	0.766	0.616	0.748	0.675
Skopje	0.563	0.345	0.632	0.631	0.437	0.509	0.638	0.125	0.417	0.212
Sofia	0.718	0.476	0.376	0.711	0.637	0.569	0.445	0.256	0.374	0.337
Stockholm	0.804	0.785	0.577	0.886	0.906	0.860	0.663	0.776	0.768	0.708
Strasbourg	0.835	0.856	0.771	0.874	0.886	0.874	0.804	0.497	0.669	0.741
Tallinn	0.747	0.576	0.650	0.872	0.849	0.787	0.613	0.769	0.694	0.787
Tirana	0.283	0.446	0.441	0.508	0.467	0.584	0.696	0.279	0.450	0.527
Turin	0.615	0.711	0.653	0.772	0.799	0.846	0.620	0.324	0.551	0.458
Tyneside conurbation	0.799	0.851	0.706	0.806	0.835	0.812	0.737	0.831	0.835	0.677
Valletta	0.450	0.685	0.443	0.336	0.430	0.420	0.661	0.344	0.448	0.539
Verona	0.563	0.815	0.654	0.794	0.732	0.748	0.725	0.454	0.565	0.683
Vilnius	0.590	0.594	0.529	0.821	0.849	0.829	0.525	0.712	0.737	0.779
Warszawa	0.763	0.387	0.659	0.846	0.833	0.735	0.627	0.475	0.466	0.661
Wien	0.950	0.848	0.642	0.944	0.895	0.872	0.721	0.844	0.746	0.867
Zagreb	0.765	0.641	0.570	0.850	0.812	0.807	0.708	0.680	0.660	0.688
Zurich	0.962	0.919	0.820	0.938	0.900	0.890	0.856	0.915	0.775	0.895

**Table A3:** Degrees of hesitancy for *IFVs* for cities.

City	C_1_	C_2_	C_3_	C_4_	C_5_	C_6_	C_7_	C_8_	C_9_	C_10_
Aalborg	0.131	0.040	0.159	0.038	0.023	0.056	0.099	0.027	0.011	0.016
Amsterdam	0.039	0.022	0.137	0.071	0.010	0.015	0.210	0.051	0.020	0.013
Ankara	0.057	0.001	0.167	0.128	0.013	0.009	0.072	0.008	0.004	0.003
Antalya	0.101	0.009	0.157	0.109	0.008	0.015	0.106	0.011	0.015	0.006
Antwerpen	0.054	0.007	0.096	0.029	0.010	0.006	0.051	0.020	0.027	0.024
Athina	0.042	0.034	0.097	0.039	0.004	0.008	0.145	0.012	0.011	0.002
Barcelona	0.049	0.013	0.094	0.067	0.009	0.021	0.125	0.011	0.004	0.016
Belfast	0.062	0.025	0.147	0.041	0.006	0.026	0.091	0.021	0.028	0.008
Beograd	0.091	0.018	0.072	0.043	0.004	0.016	0.053	0.018	0.014	0.008
Berlin	0.036	0.014	0.178	0.042	0.006	0.003	0.174	0.020	0.007	0.013
Białystok	0.118	0.036	0.110	0.036	0.005	0.008	0.135	0.011	0.005	0.010
Bologna	0.057	0.024	0.152	0.058	0.011	0.017	0.145	0.011	0.013	0.004
Bordeaux	0.023	0.008	0.077	0.034	0.017	0.013	0.082	0.014	0.006	0.010
Braga	0.151	0.011	0.086	0.060	0.012	0.011	0.053	0.015	0.002	0.004
Bratislava	0.121	0.029	0.122	0.083	0.012	0.039	0.195	0.038	0.012	0.011
Bruxelles	0.042	0.015	0.140	0.070	0.009	0.022	0.127	0.010	0.010	0.008
Bucharest	0.098	0.044	0.219	0.132	0.024	0.015	0.128	0.009	0.009	0.014
Budapest	0.065	0.030	0.230	0.098	0.012	0.013	0.234	0.005	0.014	0.003
Burgas	0.107	0.029	0.144	0.109	0.018	0.013	0.161	0.018	0.014	0.008
Cardiff	0.076	0.015	0.090	0.029	0.012	0.016	0.174	0.019	0.015	0.014
Cluj-Napoca	0.151	0.045	0.125	0.065	0.028	0.015	0.086	0.014	0.014	0.009
Diyarbakir	0.049	0.010	0.123	0.082	0.001	0.013	0.014	0.031	0.004	0.018
Dortmund	0.059	0.031	0.147	0.043	0.002	0.023	0.145	0.018	0.034	0.015
Dublin	0.025	0.013	0.062	0.009	0.006	0.020	0.070	0.012	0.005	0.009
Essen	0.092	0.015	0.144	0.044	0.014	0.014	0.164	0.024	0.017	0.006
Gdańsk	0.066	0.030	0.115	0.049	0.012	0.015	0.175	0.022	0.017	0.023
Genève	0.042	0.021	0.130	0.069	0.020	0.013	0.114	0.025	0.022	0.018
Glasgow	0.054	0.022	0.072	0.041	0.014	0.031	0.168	0.022	0.010	0.011
Graz	0.014	0.014	0.155	0.025	0.011	0.011	0.079	0.010	0.007	0.006
Groningen	0.090	0.029	0.145	0.052	0.010	0.029	0.098	0.031	0.004	0.014
Hamburg	0.021	0.037	0.180	0.027	0.008	0.012	0.178	0.019	0.026	0.009
Helsinki	0.032	0.030	0.092	0.119	0.020	0.018	0.161	0.009	0.020	0.017
Irakleio	0.127	0.012	0.088	0.050	0.008	0.017	0.106	0.006	0.000	0.018
Istanbul	0.049	0.001	0.094	0.101	0.010	0.007	0.034	0.012	0.008	0.013
København	0.042	0.052	0.215	0.065	0.017	0.050	0.197	0.040	0.015	0.009
Košice	0.076	0.024	0.100	0.059	0.007	0.034	0.130	0.032	0.022	0.010
Kraków	0.042	0.017	0.121	0.021	0.020	0.015	0.172	0.014	0.016	0.004
Lefkosia	0.219	0.039	0.185	0.091	0.014	0.016	0.095	0.040	0.005	0.010
Leipzig	0.041	0.011	0.211	0.023	0.010	0.023	0.260	0.023	0.027	0.013
Liège	0.067	0.009	0.133	0.067	0.030	0.038	0.099	0.036	0.039	0.013
Lille	0.060	0.010	0.073	0.079	0.022	0.025	0.065	0.031	0.012	0.011
Lisboa	0.089	0.035	0.153	0.098	0.037	0.016	0.135	0.047	0.008	0.016
Ljubljana	0.054	0.020	0.100	0.030	0.001	0.013	0.082	0.015	0.003	0.003
London	0.022	0.032	0.136	0.042	0.004	0.035	0.219	0.024	0.016	0.009
Luxembourg	0.029	0.017	0.090	0.069	0.013	0.027	0.122	0.016	0.014	0.000
Madrid	0.036	0.028	0.097	0.067	0.008	0.022	0.118	0.027	0.022	0.001
Málaga	0.067	0.014	0.054	0.048	0.027	0.023	0.097	0.027	0.006	0.004
Malmö	0.064	0.036	0.188	0.030	0.006	0.016	0.245	0.011	0.018	0.010
Manchester	0.068	0.024	0.128	0.053	0.022	0.022	0.195	0.026	0.022	0.018
Marseille	0.051	0.012	0.119	0.045	0.023	0.016	0.129	0.021	0.005	0.005
Miskolc	0.116	0.021	0.212	0.160	0.015	0.038	0.214	0.004	0.006	0.008
Munich	0.015	0.024	0.186	0.029	0.012	0.023	0.221	0.016	0.026	0.009
Naples	0.039	0.013	0.052	0.026	0.016	0.016	0.050	0.012	0.009	0.004
Oslo	0.014	0.026	0.122	0.030	0.006	0.029	0.173	0.015	0.014	0.009
Ostrava	0.080	0.047	0.112	0.041	0.014	0.034	0.099	0.011	0.019	0.010
Oulu	0.092	0.036	0.054	0.086	0.027	0.024	0.102	0.009	0.023	0.009
Oviedo	0.081	0.025	0.098	0.075	0.010	0.005	0.164	0.027	0.006	0.009
Palermo	0.049	0.027	0.099	0.048	0.021	0.005	0.077	0.018	0.007	0.011
Paris	0.018	0.009	0.111	0.030	0.009	0.014	0.157	0.014	0.008	0.000
Piatra Neamt	0.206	0.053	0.173	0.120	0.035	0.032	0.116	0.006	0.011	0.011
Podgorica	0.108	0.009	0.013	0.021	0.002	0.007	0.018	0.004	0.005	0.003
Praha	0.030	0.006	0.097	0.052	0.007	0.020	0.111	0.019	0.011	0.010
Rennes	0.021	0.028	0.073	0.033	0.015	0.016	0.062	0.017	0.012	0.006
Reykjavík	0.313	0.067	0.097	0.057	0.072	0.058	0.115	0.039	0.064	0.016
Riga	0.112	0.050	0.214	0.054	0.023	0.034	0.197	0.019	0.016	0.008
Rome	0.028	0.014	0.088	0.022	0.010	0.006	0.092	0.008	0.011	0.006
Rostock	0.058	0.026	0.195	0.087	0.010	0.019	0.329	0.007	0.006	0.005
Rotterdam	0.039	0.045	0.126	0.069	0.012	0.015	0.145	0.022	0.026	0.005
Skopje	0.093	0.012	0.047	0.052	0.004	0.001	0.032	0.000	0.004	0.002
Sofia	0.094	0.030	0.262	0.093	0.022	0.045	0.238	0.041	0.017	0.009
Stockholm	0.032	0.008	0.209	0.016	0.001	0.028	0.205	0.020	0.014	0.009
Strasbourg	0.039	0.016	0.084	0.062	0.008	0.016	0.083	0.017	0.007	0.003
Tallinn	0.120	0.036	0.215	0.051	0.028	0.038	0.285	0.016	0.016	0.011
Tirana	0.046	0.009	0.015	0.041	0.000	0.003	0.026	0.001	0.001	0.004
Turin	0.041	0.017	0.168	0.128	0.015	0.009	0.178	0.008	0.008	0.000
Tyneside conurbation	0.088	0.010	0.136	0.058	0.021	0.036	0.185	0.026	0.019	0.013
Valletta	0.249	0.091	0.204	0.151	0.016	0.045	0.229	0.008	0.008	0.006
Verona	0.119	0.014	0.107	0.050	0.010	0.007	0.141	0.012	0.014	0.009
Vilnius	0.216	0.032	0.211	0.091	0.025	0.011	0.247	0.024	0.018	0.009
Warszawa	0.091	0.045	0.194	0.083	0.013	0.017	0.219	0.031	0.013	0.014
Wien	0.004	0.011	0.183	0.011	0.005	0.025	0.139	0.006	0.017	0.003
Zagreb	0.030	0.020	0.086	0.048	0.006	0.016	0.048	0.027	0.012	0.018
Zurich	0.005	0.018	0.128	0.029	0.007	0.003	0.091	0.013	0.025	0.004

**Table A4:** Values of *IFT* coefficients for cities.

City	*IFT* Coefficient	City	*IFT* Coefficient	City	*IFT* Coefficient	City	*IFT* Coefficient
Aalborg	0.837	Diyarbakir	0.598	Ljubljana	0.786	Reykjavík	0.747
Amsterdam	0.776	Dortmund	0.786	London	0.737	Riga	0.699
Ankara	0.609	Dublin	0.764	Luxembourg	0.839	Rome	0.470
Antalya	0.667	Essen	0.729	Madrid	0.571	Rostock	0.769
Antwerpen	0.798	Gdańsk	0.752	Málaga	0.658	Rotterdam	0.793
Athina	0.434	Genève	0.809	Malmö	0.772	Skopje	0.468
Barcelona	0.637	Glasgow	0.794	Manchester	0.766	Sofia	0.528
Belfast	0.795	Graz	0.764	Marseille	0.603	Stockholm	0.781
Beograd	0.545	Groningen	0.850	Miskolc	0.639	Strasbourg	0.772
Berlin	0.711	Hamburg	0.785	Munich	0.813	Tallinn	0.751
Białystok	0.784	Helsinki	0.836	Naples	0.421	Tirana	0.477
Bologna	0.695	Irakleio	0.524	Oslo	0.800	Turin	0.643
Bordeaux	0.803	Istanbul	0.548	Ostrava	0.700	Tyneside conurbation	0.804
Braga	0.778	København	0.757	Oulu	0.786	Valletta	0.523
Bratislava	0.578	Košice	0.666	Oviedo	0.779	Verona	0.684
Bruxelles	0.673	Kraków	0.642	Palermo	0.377	Vilnius	0.718
Bucharest	0.490	Lefkosia	0.616	Paris	0.649	Warszawa	0.658
Budapest	0.604	Leipzig	0.774	Piatra Neamt	0.703	Wien	0.831
Burgas	0.654	Liège	0.683	Podgorica	0.594	Zagreb	0.725
Cardiff	0.821	Lille	0.749	Praha	0.744	Zurich	0.886
Cluj-Napoca	0.667	Lisboa	0.615	Rennes	0.846	-	-

**Table A5:** Classification of cities with respect to the level of quality of life.

City	Level	City	Level	City	Level	City	Level
Aalborg	high	Diyarbakir	medium	Ljubljana	high	Reykjavík	medium
Amsterdam	high	Dortmund	high	London	high	Riga	medium
Ankara	medium	Dublin	high	Luxembourg	high	Rome	medium
Antalya	medium	Essen	high	Madrid	medium	Rostock	high
Antwerpen	high	Gdańsk	high	Málaga	medium	Rotterdam	high
Athina	low	Genève	high	Malmö	high	Skopje	medium
Barcelona	medium	Glasgow	high	Manchester	high	Sofia	medium
Belfast	high	Graz	high	Marseille	medium	Stockholm	high
Beograd	medium	Groningen	high	Miskolc	medium	Strasbourg	high
Berlin	medium	Hamburg	high	Munich	high	Tallinn	medium
Białystok	high	Helsinki	high	Naples	low	Tirana	low
Bologna	medium	Irakleio	medium	Oslo	high	Turin	medium
Bordeaux	high	Istanbul	medium	Ostrava	high	Tyneside conurbation	high
Braga	high	København	high	Oulu	high	Valletta	medium
Bratislava	medium	Košice	medium	Oviedo	high	Verona	medium
Bruxelles	medium	Kraków	medium	Palermo	low	Vilnius	medium
Bucharest	medium	Lefkosia	medium	Paris	medium	Warszawa	medium
Budapest	medium	Leipzig	high	Piatra Neamt	medium	Wien	high
Burgas	medium	Liège	medium	Podgorica	medium	Zagreb	high
Cardiff	high	Lille	high	Praha	high	Zurich	high
Cluj-Napoca	medium	Lisboa	medium	Rennes	high	-	-

**Table A6:** Values of *IFT* coefficients for cities.

City	*IFT* Coefficient	City	*IFT* Coefficient	City	*IFT* Coefficient	City	*IFT* Coefficient
Aalborg	0.698	Diyarbakir	0.530	Ljubljana	0.667	Reykjavík	0.596
Amsterdam	0.637	Dortmund	0.639	London	0.616	Riga	0.553
Ankara	0.517	Dublin	0.657	Luxembourg	0.690	Rome	0.430
Antalya	0.567	Essen	0.601	Madrid	0.491	Rostock	0.618
Antwerpen	0.663	Gdańsk	0.609	Málaga	0.561	Rotterdam	0.650
Athina	0.386	Genève	0.665	Malmö	0.631	Skopje	0.412
Barcelona	0.533	Glasgow	0.669	Manchester	0.633	Sofia	0.427
Belfast	0.669	Graz	0.674	Marseille	0.514	Stockholm	0.635
Beograd	0.466	Groningen	0.704	Miskolc	0.507	Strasbourg	0.649
Berlin	0.598	Hamburg	0.647	Munich	0.683	Tallinn	0.590
Białystok	0.648	Helsinki	0.673	Naples	0.399	Tirana	0.392
Bologna	0.576	Irakleio	0.452	Oslo	0.660	Turin	0.543
Bordeaux	0.666	Istanbul	0.480	Ostrava	0.602	Tyneside conurbation	0.664
Braga	0.602	København	0.622	Oulu	0.633	Valletta	0.426
Bratislava	0.481	Košice	0.556	Oviedo	0.625	Verona	0.563
Bruxelles	0.552	Kraków	0.545	Palermo	0.366	Vilnius	0.554
Bucharest	0.409	Lefkosia	0.499	Paris	0.558	Warszawa	0.531
Budapest	0.493	Leipzig	0.628	Piatra Neamt	0.555	Wien	0.708
Burgas	0.550	Liège	0.554	Podgorica	0.523	Zagreb	0.607
Cardiff	0.688	Lille	0.616	Praha	0.631	Zurich	0.758
Cluj-Napoca	0.554	Lisboa	0.494	Rennes	0.693	-	-
